# Supplementary material for: Hierarchical Porous Heteroatoms—Co-Doped Activated Carbon Synthesized from Coconut Shell and Its Application for Supercapacitors
Source: Nanomaterials (Basel). 2022 Oct 7;12(19):3504. doi: 10.3390/nano12193504 (PMC9565498; doi:10.3390/nano12193504)
Supplement: Supplementary file 1 [file nanomaterials-12-03504-s001.zip › nanomaterials-1936275-supplementary.pdf]

nanomaterials

MDPI

Supplementary Materials

Hierarchical Porous Heteroatoms—Co-Doped Activated Carbon Synthesized from Coconut Shell and Its Application for Supercapacitors

Rui Liu [1], Jing-Xuan Wang [2] and Wein-Duo Yang [2,*]

[1] Center of Pharmaceutical Engineering and Technology, School of Pharmacy, Harbin University of Commerce, Harbin 150076, China
[2] Department of Chemical and Materials Engineering, National Kaohsiung University of Science and Technology, Kaohsiung 80778, Taiwan
* Correspondence: ywd@nkust.edu.tw; Tel: +886-73-81-4526 (ext. 15116)

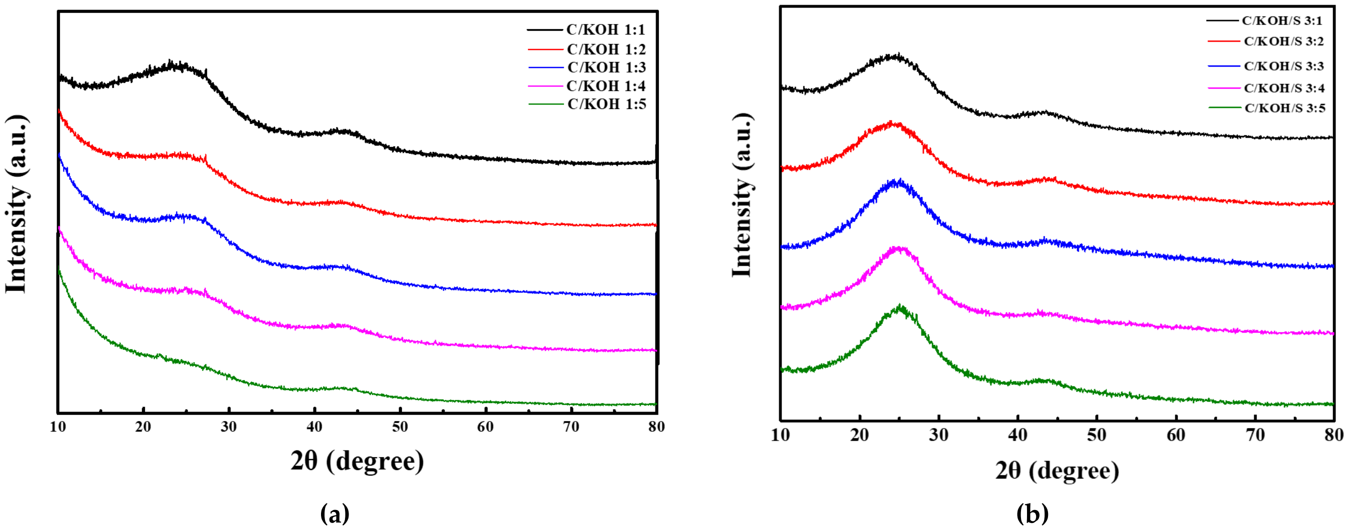

Figure S1. XRD patterns of the as-obtained porous activated carbon.

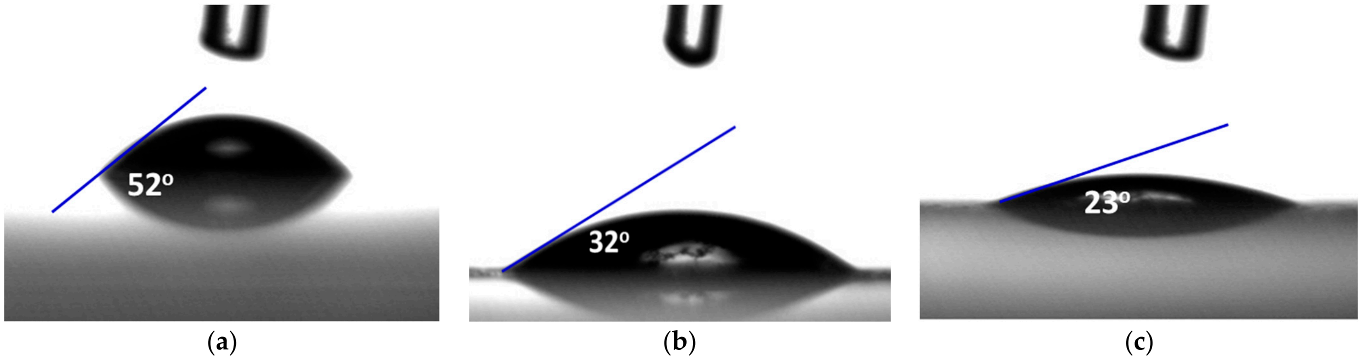

Figure S2. The water contact angle of the as-obtained porous carbon coated on glass slide: (**a**) water on glass slide; (**b**) C/KOH-3 sample; (**c**) C/KOH/S:3:3-700 sample.

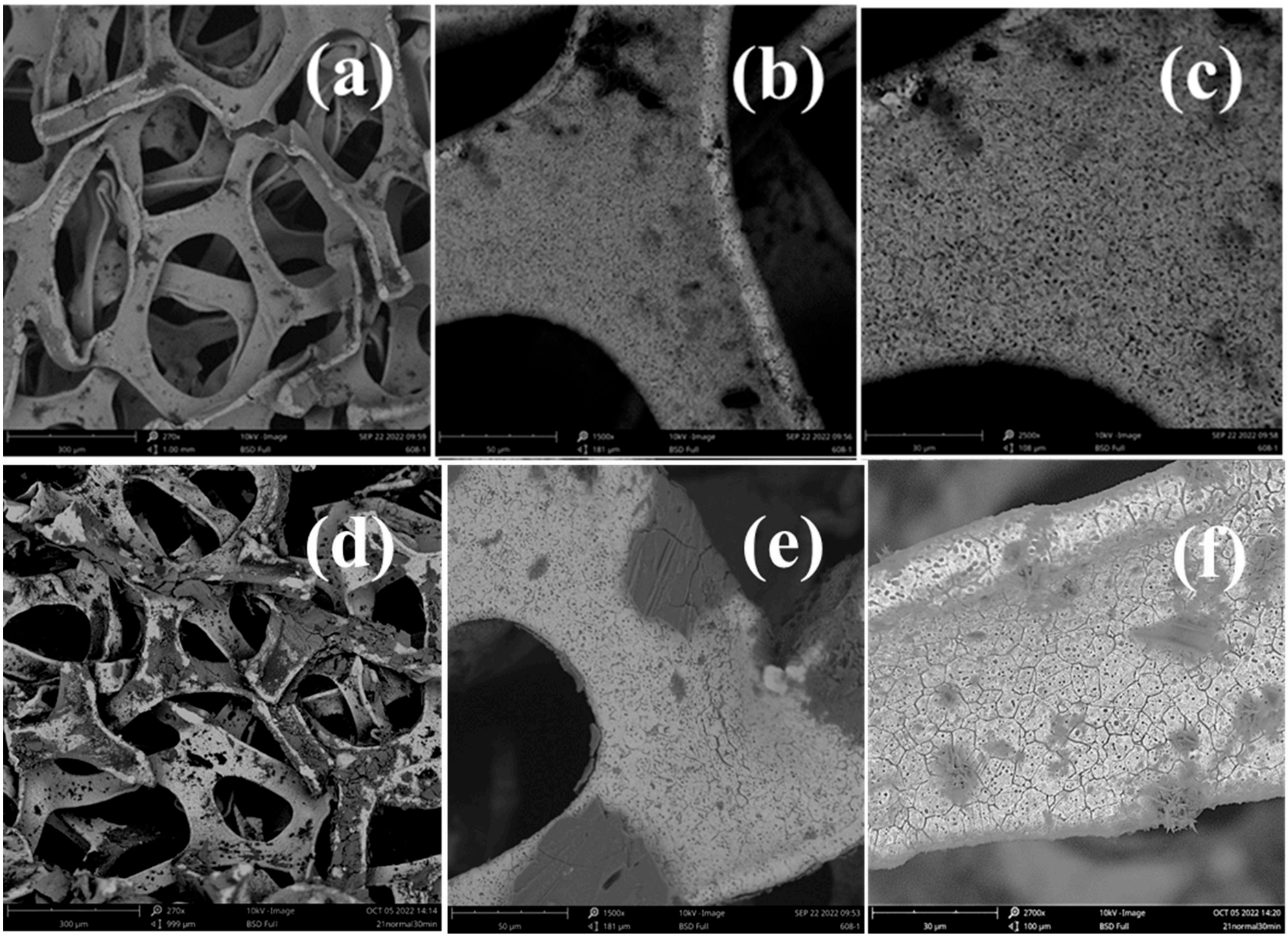

Figure S3. The C/KOH/S:3:3-700 electrode examined by SEM before/after 1000 charge/discharge cycles test. Before test on magnification at 270, 1500, and 2500, respectively(**a**–**c**); after test on magnification at 270, 1500, and 2500, respectively (**d**–**f**).
